# Chronic Hyperglycemia Drives Functional Impairment of Lymphocytes in Diabetic *INS*^C94Y^ Transgenic Pigs

**DOI:** 10.3389/fimmu.2020.607473

**Published:** 2021-01-22

**Authors:** Isabella-Maria Giese, Marie-Christin Schilloks, Roxane L. Degroote, Maria Weigand, Simone Renner, Eckhard Wolf, Stefanie M. Hauck, Cornelia A. Deeg

**Affiliations:** ^1^Chair of Physiology, Department of Veterinary Sciences, LMU Munich, Munich, Germany; ^2^Chair for Molecular Animal Breeding and Biotechnology, Gene Center and Department of Veterinary Sciences, LMU Munich, Munich, Germany; ^3^Center for Innovative Medical Models (CiMM), Department of Veterinary Sciences, LMU Munich, Munich, Germany; ^4^German Center for Diabetes Research (DZD), Neuherberg, Germany; ^5^Laboratory for Functional Genome Analysis (LAFUGA), Gene Center, LMU Munich, Munich, Germany; ^6^Research Unit Protein Science, Helmholtz Center Munich, German Research Center for Environmental Health GmbH, Munich, Germany

**Keywords:** animal model(s), annexin A1, proliferation, diabetes mellitus, cell metabolism, PHA, T cells, impaired immune cell function

## Abstract

People with diabetes mellitus have an increased risk for infections, however, there is still a critical gap in precise knowledge about altered immune mechanisms in this disease. Since diabetic *INS*^C94Y^ transgenic pigs exhibit elevated blood glucose and a stable diabetic phenotype soon after birth, they provide a favorable model to explore functional alterations of immune cells in an early stage of diabetes mellitus *in vivo*. Hence, we investigated peripheral blood mononuclear cells (PBMC) of these diabetic pigs compared to non-diabetic wild-type littermates. We found a 5-fold decreased proliferative response of T cells in *INS*^C94Y^ tg pigs to polyclonal T cell mitogen phytohemagglutinin (PHA). Using label-free LC-MS/MS, a total of 3,487 proteins were quantified, and distinct changes in protein abundances in CD4^+^ T cells of early-stage diabetic pigs were detectable. Additionally, we found significant increases in mitochondrial oxygen consumption rate (OCR) and higher basal glycolytic activity in PBMC of diabetic *INS*^C94Y^ tg pigs, indicating an altered metabolic immune cell phenotype. Thus, our study provides new insights into molecular mechanisms of dysregulated immune cells triggered by permanent hyperglycemia.

## Introduction

Diabetes mellitus is a major risk factor regarding the outcome of bacterial and viral infections (1). Among diabetic patients, increased numbers of highly susceptible individuals suffering from tuberculosis (2) or influenza (3) are common. Recently, meta-analyses revealed an enhanced severity and mortality of COVID-19 infections in people with diabetes mellitus (4, 5). Undoubtedly, there is a link between the disease and an impaired immune system, but the molecular mechanisms involved in altered immune cell function in diabetic patients are still unknown. Dysfunctional T cells may play a pivotal role in immunological impairment in diabetes mellitus (6). However, the precise effects of chronic hyperglycemia on immune cell function are still uncertain since contradictory findings of both hyperresponsive (7) and attenuated (8, 9) T cells were reported in type 2 diabetic patients. While numerous studies linked hyperglycemia to enhanced T cell activation and proliferation (7), others suggest that an abnormal glucose homeostasis promotes an insufficient T-cell response *via* an increased frequency of senescent T cells with proliferative impairment (8, 9). Thus, it has yet to be determined whether dysfunctional T cells are impaired or highly activated in diabetes mellitus and whether hyperglycemia causes immune cell dysregulation.

Alterations of cellular mechanisms and molecular networks in disease states can be analysed by changes in proteome composition (10). Still little is known about accurate protein profiles of T cells affected by hyperglycemia *in vivo*. Therefore, mass spectrometry-based studies are a powerful tool for obtaining comprehensive information regarding cellular pathology (11) and enable hypothesis-generating approaches on the protein level to understand the molecular basis of altered proteome composition and regulation caused by disease. Moreover, recent advances in the field of immuno-metabolism recognized cellular metabolism as a primary driver and regulator of immune cell function, leading to a wide variety of functionally different immune cells (12). Since there is still a lack of knowledge about hyperglycemia-associated alterations of immune cell metabolic phenotypes, there is an urgent need to understand whether and how high glucose levels affect the metabolic phenotype of the cells and thereby change their immune function (12).

Diabetic *INS*^C94Y^ transgenic (tg) pigs were generated as a large animal model of permanent neonatal diabetes mellitus (13). These pigs are characterized by impaired insulin secretion with consecutive hypoinsulinemia and increased fasting blood glucose levels (13). Early on, *INS*^C94Y^ tg pigs show a stable diabetic phenotype and mirror several disease-associated alterations of diabetes mellitus as seen in humans such as cataract, retinopathy, impaired myocardial function and regeneration (14, 15). Since early stages of disease in diabetic patients are often unperceived for a long time, initial immunological alterations are difficult to observe due to the latent disease process. Thus, this diabetic pig model enables the exploration of deviant immune cell function and underlying mechanisms in an early stage of diabetes mellitus with hyperglycemia and hypoinsulinemia, offering valuable insights into disease pathogenesis. Hence, we used PBMC of young *INS*^C94Y^ tg pigs and non-diabetic wild-type littermates to investigate the impact of a permanent early-life hyperglycemic condition on immune cell function.

## Materials and Methods

### Animal Model and Sample Preparation

**Figure 1:**
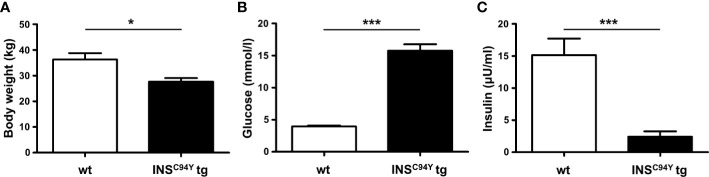
Body weight, blood glucose and serum insulin levels of *INS*^C94Y^ tg pigs (*INS*^C94Y^ tg) and wild-type littermates (wt) aged 12 weeks. **(A)** *INS*^C94Y^ tg pigs (n=16) revealed a significantly (*p < 0.05) lower body weight compared to wild-types (n=18). **(B)** Blood glucose levels showed significant differences (***p < 0.001) between the wild-type (n=26) and diabetic group (n=22) with elevated glucose levels in *INS*^C94Y^ tg pigs. **(C)** Serum insulin levels were significantly (***p < 0.001) reduced in these diabetic pigs (n=16) compared to their non-transgenic wild-type littermates (n=17).

In this study, PBMC of 23 sex-matched diabetic *INS*^C94Y^ tg pigs and 31 non-transgenic wild-type littermates at the age of 12 weeks were used. *INS*^C94Y^ tg pigs were generated previously as described (13). First, young pigs were weighed. For sampling, pigs were fasted overnight and heparinized venous whole blood was collected. Blood glucose levels were determined immediately using a Precision Xceed glucometer with Precision XtraPlus test strip (Abbott) and insulin levels were measured by an enzyme-linked immunosorbent assay (ELISA) for humans in serum (Mercodia, Uppsala, Sweden). Species reactivity with porcine insulin was specified as 93% by the manufacturer. Values below the quantification limit of the assay (≤ 1 mU/L) were arbitrarily set to 0. Body weight, blood glucose levels and serum insulin levels were in accordance with values from earlier characterizations of this large animal model (13). Due to the lack of insulin as growth factor and anabolic hormone, *INS*^C94Y^ tg pigs exhibit a significantly (*p < 0.05) lower body weight (27.7 ± 5.4 kg) than wild-type littermates (34.9 ± 10.1 kg; **Figure 1A**). Blood glucose levels were significantly (***p < 0.001) elevated in diabetic pigs (15.8 ± 4.7 mmol/L) compared to wild-types (4 ± 0.7 mmol/L; **Figure 1B**) and serum insulin levels of *INS*^C94Y^ tg pigs (6.47 ± 1.08 mU/L) were significantly reduced (***p < 0.001) compared to non-transgenic wild-types littermates (17.18 ± 9.54 mU/L; **Figure 1C**). All animals were housed under controlled conditions, had free access to water and were fed a commercial diet. PBMC were isolated by density gradient centrifugation (RT, 500 x g, 25 min, brake off) with Pancoll separating solution (PAN-Biotech, Aidenbach, Germany), and restored in PBS (pH 7.4) or RPMI medium (PAN-Biotech, Aidenbach, Germany), supplemented with 10% heat-inactivated fetal calf serum (FCS) and 1% penicillin/streptomycin (both Biochrom, Berlin, Germany). Blood withdrawal was performed according to the German Animal Welfare Act with permission from the responsible authority (Government of Upper Bavaria), following the ARRIVE guidelines and Directive 2010/63/EU. Approval numbers: AZ 55.2-1-54-2532-163-2014 and ROB-55.2-2532.Vet_02-19-195.

### Lymphocyte Stimulation by Mitogens

To assess the proliferative response to three different mitogens, five independent experiments with a total of 29 animals (wt n=15, *INS*^C94Y^ tg n=14) were performed. Duplicates (technical replicates) were generated for each animal. Mean values of technical replicates were then used for further statistical analysis. In addition to unstimulated controls, PBMC were either stimulated by pokeweed mitogen (PWM; 1 µg/mL), concanavalin A (ConA; 1 µg/mL) or phytohaemagglutinin (PHA; 1 µg/mL). After an incubation period of 32 h at 37°C, cells were incorporated with ^3^H-thymidine (Perkin Elmer, Hamburg, Germany) and incubated for 16 more h. After harvesting, ^3^H-thymidine incorporation was quantified by detecting counts per minute (cpm), using a Microbeta (Perkin Elmer, Hamburg, Germany). Proliferation rate was expressed as the ratio of ^3^H-thymidine incorporation by stimulated cells with respect to unstimulated cells.

### Flow Cytometry

Seven independent experiments with a total of 20 animals (wt n=11, *INS*^C94Y^ tg n=9) were performed. Staining of 2 x 10^5^ cells per well was performed with mouse anti-human CD79a (clone HM57, Bio-Rad AbD Serotec, Puchheim, Germany, 1:100) for identification of B cells and Alexa Fluor 647-conjugated rat anti-human CD3ϵ [clone CD3-12, 1:200; cross-reactive to pig (16)] for identification of T cells. We used FITC-conjugated mouse anti-pig CD4α (clone MIL17, Bio-Rad AbD Serotec, Puchheim, Germany, 1:20) and Alexa Fluor 647-conjugated mouse anti-pig CD8α (clone 76-2-11, Becton Dickinson, Heidelberg, Germany, 1:400) to identify αβ T cells and mouse anti-pig SWC5 (clone b37c10, Bio-Rad AbD Serotec, Puchheim, Germany, 1:100) for identification of γδ T cell subpopulation. If necessary, a secondary antibody was used (Alexa Fluor 647-conjugated goat F(ab’)2 anti-mouse IgG (Fc), Dianova, Hamburg, Germany, 1:1000). Dead cells were excluded *via* labeling with Viobility 405/520 Fixable Dye (Miltenyi Biotec, Bergisch Gladbach, Germany) prior to the various stainings mentioned above. Analyses were performed with MACSQuant Analyzer 10 and Flowlogic Software (both Miltenyi Biotec, Bergisch Gladbach, Germany).

### Magnetic Activated Cell Sorting for CD4^+^ T Cells

Briefly, a total of 6 x 10^7^ cells was incubated in staining buffer with mouse anti-pig CD4α (clone MIL17, Bio-Rad AbD Serotec, Puchheim, Germany, 1:50) at 4°C for 20 min. Staining buffer contained phosphate-buffered saline (pH 7.2) and was supplemented with 2 mM EDTA and 0.5% bovine serum albumin (BSA). In the next step, cells were resuspended in 480 μl staining buffer before adding 120 μl anti-mouse IgG_2a/b_ MicroBeads (Miltenyi Biotec, Bergisch Gladbach, Germany) for an incubation period of 15 min. In further steps, BSA in staining buffer was omitted to prevent interference with mass spectrometry. Magnetic separation was performed using LS columns (Miltenyi Biotec, Bergisch Gladbach, Germany). Magnetically-labelled CD4^+^ T cells were retained in the magnetic field, while unwanted cells were eliminated by three washing steps. Positive CD4^+^ T cell fraction was eluted by removing the column from magnetic field and flushing with staining buffer. 6 x 10^5^ positive selected cells were pelleted and stored at −20°C until filter-aided sample preparation (FASP). The isolation of porcine CD4^+^ T cells routinely achieved > 90% purity, confirmed by flow cytometry.

### Mass Spectrometry and Data Analysis

Purified CD4^+^ T cells of four diabetic *INS*^C94Y^ tg pigs and five littermate wild-types were analyzed. Samples were temporarily frozen after preparation until mass spectrometry could be performed. CD4^+^ T cell pellets were lysed in urea buffer (8M in 0.1M Tris/HCl pH 8.5) and 10 μg total protein of each sample was proteolyzed with LysC and trypsin by a modified filter-aided sample preparation (FASP) as described (17). Acidified eluted peptides were analyzed in the data-independent acquisition mode on a Q Exactive HF-X mass spectrometer (Thermo Fisher Scientific, Waltham, MA, USA) online coupled to an ultra-high-performance liquid chromatography (UHPLC) system (Ultimate 3000, Thermo Fisher Scientific). The spectral library was generated directly in Spectronaut Pulsar X (Biognosys, Schlieren, Switzerland; version 12.0.20491.17.25792) as described (18). Spectronaut was equipped with the Ensembl Pig Database (Release 75 (Sscrofa10.2), 25,859 sequences, https://www.ensembl.org). Peptide identification was filtered to satisfy an FDR of 1% by the mProphet approach with q-value cut-off at 0.05 (19).

For subsequent data evaluation, statistical analysis was performed on log2 transformed normalized abundance values using Student’s *t*-test. Differences in protein abundance with p < 0.05 were considered significant. Among all differentially abundant proteins, those with *INS*^C94Y^/wt ratio ≥ 2.0 were considered for possible biological relevance and used in further experiments. The heatmap of hierarchical cluster analysis was created with open source software Cluster 3.0 and was illustrated *via* Java TreeView (version 1.1.6r4, http://jtreeview.sourceforge.net). GraphPad Prism Software (version 5.04) was used to design Volcano plot, and pathway enrichment analysis was done with open source software Reactome (Pathway Browser version 3.7, Reactome database release 74, https://reactome.org).

### Quantification of ANXA1 by Immunofluorescence Staining

Six experiments with a total of twelve animals (wt n=6, *INS*^C94Y^ tg n=6) were performed to quantify ANXA1 in porcine PBMC. Flow cytometric analysis was performed using rabbit anti-human ANXA1 (Thermo Fisher Scienific, Ulm, Germany, 1:100) and Alexa Fluor 647-conjugated goat anti-rabbit IgG H+L (Invitrogen, Karlsruhe, Germany; 1:500). Cross reactivity of anti-human ANXA1 antibody (Invitrogen, Catalog Nr. 71-3400, 1:100) was examined by western blot. Porcine Annexin A1 was detected at ~36 kDa and human Annexin A1 was detected at ~30 and ~36 kDa. In addition, sequence homology of porcine ANXA1 was confirmed *via* BLASTP (https://blast.ncbi.nlm.nih.gov/Blast.cgi). Amino acid sequences of human Annexin A1 (P04083) and Sus scrofa Annexin A1 (P19619) were blasted against each other and resulted in 307/346 identities (88.73%). For extra- and intracellular staining, PBMC were permeabilized (BD Cytofix/Cytoperm, Becton Dickinson, Heidelberg, Germany) for 20 min at 4°C and washed with washing buffer (BD Perm/Wash, Becton Dickinson, Heidelberg, Germany), diluted in PBS 1:10.

For immunofluorescence staining, purified CD4^+^ T cells of wild-type and *INS*^C94Y^ tg pigs were incubated with FITC-conjugated anti-pig CD4α before staining of ANXA1. After fixation with 1% PFA, cell nuclei were counterstained with 4′,6-diamidino-2-phenylindole (DAPI; Invitrogen, Karlsruhe, Germany, 1:100) for 30 min at RT. 5 x 10^4^ cells were transferred to microscope slides and centrifugated (300 x g, 10 min) before coverslip using mounting medium (Serva, Rosenheim, Germany). Visualization of stained targets was performed using a Leica Dmi8 microscope with associated LAS-X-software (Leica, Wetzlar, Germany).

### Measurement of Oxygen Consumption Rate and Extracellular Acidification Rate by Seahorse XFe Analyzer

Metabolic phenotypes of PBMC from 15 wild-types and 10 *INS*^C94Y^ tg pigs were determined in five independent experiments, using a Seahorse XFe Analyzer (Agilent Technologies, Waldbronn, Germany) measuring oxygen consumption rate (OCR), which is attributed to mitochondrial respiration and extracellular acidification rate (ECAR), which can be related to glycolysis (20). Duplicates (technical replicates) were generated for each animal. Mean values of technical replicates were then used for further statistical analysis. In accordance with the manufacturer’s instructions, sterile XF assay buffer (Seahorse XF RPMI Medium supplemented with 10 mM glucose, 2 mM L-glutamine, and 1 mM pyruvate, pH 7.4; Agilent Technologies, Waldbronn, Germany) was used for experiments. Prior to the start of the assay, sensor cartridges (Agilent Technologies, Waldbronn, Germany) were prepared adding oligomycin, FCCP and rotenone & antimycin A. A total of 1 x 10^6^ PBMC was seeded in 24-well XF24 cell culture microplates (Agilent Technologies, Waldbronn, Germany), while four wells were kept free from cells as background correction. Baseline OCR and ECAR were measured before adding oligomycin, FCCP and rotenone & antimycin A. OCR was reported in units of pmol/minute and ECAR in mpH/minute.

### Statistical Analysis

Kolmogorow-Smirnov (KS) test was performed for determination of Gaussian distribution. If KS test indicated p < 0.05 (no normal distribution), Mann-Whitney U test was used for statistical analysis, while Student’s *t*-test was used if KS test was p > 0.05 (normal distribution). In both tests, statistical probabilities were considered significant at p < 0.05. Significances are indicated by asterisks with *p < 0.05, **p < 0.01, and ***p < 0.001.

## Results

### Reduced Proliferative Capacity of Lymphocytes in Diabetic *INS*^C94Y^ Transgenic Pigs After Polyclonal Stimulation With Phytohemagglutinin

**Figure 2:**
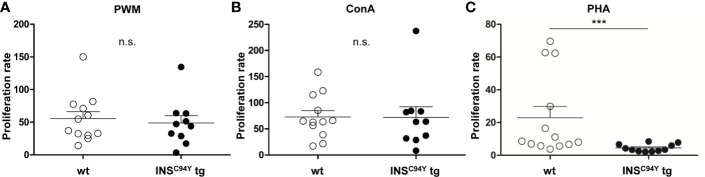
Proliferative capacity of porcine lymphocytes. Mitogens were used for stimulation of lymphocytes analyzed after 48 h of culture: **(A)** pokeweed mitogen (PWM) **(B)** concanavalin A (ConA) or **(C)** phytohaemagglutinin (PHA). Proliferation rate of stimulated lymphocytes did not differ between wild-type (wt, n=12) and *INS*^C94Y^ tg pigs (n=10) stimulated by ConA and PWM (ns = not significant). After PHA-stimulation, lymphocytes of *INS*^C94Y^ tg pigs (n=11) revealed a significantly 5-fold decreased proliferation rate (***p < 0.001) compared to non-transgenic wild-type littermates (n=13). Data are represented as means +SEM.

For assessment of the proliferation response *in vitro*, we used B and T cell mitogen PWM (21) and the specific T cell mitogens ConA (22) and PHA (23) to stimulate lymphocytes of diabetic *INS*^C94Y^ tg pigs and wild-type littermates. Interestingly, while no differences could be observed between the two groups after stimulation with PWM (**Figure 2A**) and ConA (**Figure 2B**), lymphocytes of diabetic pigs (n=11) revealed a significantly 5-fold decreased proliferation rate in response to PHA compared to wild-types (n=13) (**Figure 2C**; ***p < 0.001).

### Lymphocyte Subpopulation Ratio Did Not Differ Between Wild-Types and Diabetic Pigs

Next, we examined whether the decreased ability to proliferate correlated with altered leucocyte populations in diabetic *INS*^C94Y^ tg pigs. Relative numbers of peripheral blood mononuclear cells were counted *via* DiffQuick stained blood smears. Wild-type (n=6) and *INS*^C94Y^ tg pigs (n=6) displayed no significant difference in relative PBMC counts (**Supplementary Figure 1**). Next, lymphocyte subpopulations were examined (**Supplementary Figure 2**). There were no significant differences in the percentage of B or T cell population between wild-types and diabetic pigs (**Supplementary Figures 2A, B**). Moreover, measurements of T cell subsets CD4^+^ (**Supplementary Figure 2C**) and CD8α^+^ (**Supplementary Figure 2D**) as well as pig-specific CD4^+^CD8α^+^ double positive αβ T cells (**Supplementary Figure 2E**) and SWC5^+^ γδ T cells (**Supplementary Figure 2F**) revealed no differences between both groups.

### CD4^+^ T Cells of Diabetic *INS*^C94Y^ Transgenic Pigs Showed Divergent Proteome Profile Which Associates to Metabolic Pathways

**Figure 3:**
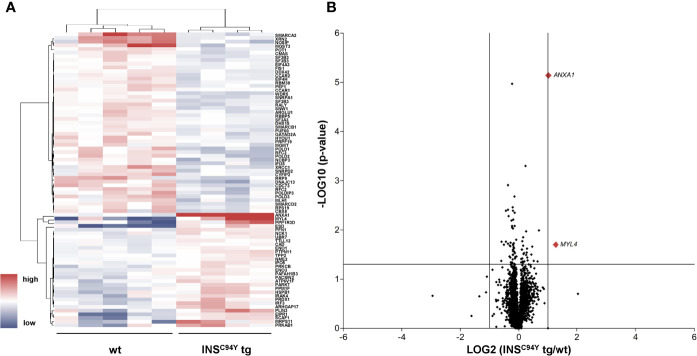
Quantitative proteome analyses of porcine CD4^+^ T cells. **(A)** Hierarchical cluster analysis of the porcine CD4^+^ T cell proteome revealed significant differences in protein abundance (p < 0.05) in *INS*^C94Y^ tg pigs (n=4) and non-transgenic littermates (wt, n=5) indicated by their human orthologue gene names. Red color indicates increased and blue color decreased abundances of the identified proteins. **(B)** Volcano plot illustrates quantified proteins (≥ 2 unique peptides) in CD4^+^ T cells of *INS*^C94Y^ tg pigs and wild-types (wt). Log2 abundance differences between groups (x-axis) are plotted against Log10 statistical significance values (y-axis). The horizontal line indicates the p-value cut-off at 0.05, the vertical line shows ratio cut-off at 2. The two proteins that satisfied the biological (ratio ≥ 2) and statistical (p < 0.05) significance threshold are shown in red (MYL4 and ANXA1).

In order to gain deeper insights into the observed proliferation on molecular level, we characterized the proteomes of porcine CD4^+^ T cells, which we hypothesized to be the key drivers of impaired proliferation response. Purified CD4^+^ T cells were examined with differential proteome analyses, using data-independent acquisition LC−MS/MS. A high-resolution proteome was obtained, with a total of 3,487 identified proteins, of which 2,704 were quantified with at least two unique peptides (**Supplementary Table 1**). Among these, 80 proteins were significantly different in abundance (*p < 0.05) (**Supplementary Table 2**, **Figure 3A**).

Pathway analysis of these 80 proteins *via* the Reactome database (24) revealed that enriched pathways differed substantially between groups (**Supplementary Table 3**, **Supplementary Figure 3**). Most significantly enriched pathways in cells from wild-types mainly associated to processes involved in DNA repair, cell cycle and chromatin remodeling (**Table 1**, **Supplementary Figure 3A**), whereas cells from *INS*^C94Y^ tg pigs predominantly associated to signal transduction, immune system and metabolic pathways (**Table 2**, **Supplementary Figure 3B**). Among the latter, lipophagy was one of the most significantly enriched pathways (**Table 2**). This is especially interesting, since lipophagy is linked to metabolic dysfunction of immune cells (25, 26), and it was recently shown that functionally different cell subtypes can be identified solely by their metabolic phenotype (12).

**Table 1:** Pathway enrichment analysis of CD4^+^ T cells from wild-type littermates.

Most significant pathways enriched in CD4^+^ T cells of wild-types		
Pathway name	p-value	Identified proteins
Gap-filling DNA repair synthesis and ligation in GG-NER	2.26e-13	XRCC1, POLD1,POLD2, POLD3, RFC2, RFC3
Polymerase switching	5.14e-13	POLD1,POLD2, POLD3, RFC2, RFC3
Leading Strand Synthesis	5.14e-13	RFC2, POLD2, POLD1, POLD3, RFC3

**Table 2:** Pathway enrichment analysis of CD4^+^ T cells from *INS*^C94Y^ tg pigs.

Most significant pathways enriched in CD4^+^ T cells of *INS*^C94Y^ tg pigs		
Pathway name	p-value	Identified proteins
RUNX1 regulates transcription of genes involved in differentiation of myeloid cells	4.5e-04	PRKCB
Lipophagy	5.34e-04	PLIN3, PRKAB1
Activation of IRF3/IRF7 mediated by TBK1/IKK epsilon	1.46e-03	PTPN11, IRF3

### Increased Basal Glycolytic Activity and Mitochondrial Respiration in Peripheral Blood Mononuclear Cells of Diabetic Pigs

**Figure 4:**
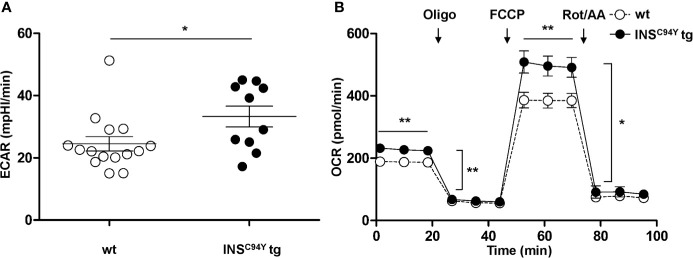
Basal glycolytic and mitochondrial respiratory profiles of porcine PBMC. **(A)** Basal glycolytic rate (Extracellular acidification rate, ECAR) was significantly elevated in PBMC of *INS*^C94Y^ tg pigs (n=10) compared to wild-type littermates (wt, n=15) (*p < 0.05). **(B)** Oxygen consumption rate (OCR) was measured under basal conditions and after injection of oligomycin, FCCP, rotenone and antimycin. Compared to PBMC from wild-types (wt, n=15), mitochondrial respiration of PBMC from diabetic pigs (n=10) yielded a significantly increased basal, ATP-linked and maximal respiration (**p < 0.01) as well as a significantly increased spare respiratory capacity (*p < 0.05). Data are represented as means ± SEM.

Since Reactome pathway analysis revealed association to metabolic pathways, especially lipophagy (**Table 2**), we were interested whether the metabolic phenotype of PBMC from *INS*^C94Y^ tg pigs differs from wild-types. Interestingly, the basal glycolytic rate of PBMC from diabetic pigs was significantly higher compared to wild-type PBMC (**Figure 4A**; *p < 0.05), indicating increased lactate production and glycolytic activity of diabetic PBMC. Furthermore, analyses of OCR revealed a significant increase in oxygen consumption at all measured time points in diabetic pigs. Compared to wild-type PBMC, a rise of basal, ATP-linked and maximal mitochondrial respiration (**p < 0.01) as well as spare respiratory capacity (*p < 0.05) was present in PBMC from diabetic pigs (**Figure 4B**). These differences indicated a fundamentally different metabolic phenotype of PBMC from wild-types (n=15) and diabetic *INS*^C94Y^ tg pigs (n=10).

### Diabetes-Associated Protein ANXA1 Was Highly Abundant in CD4^+^ T Cells of Diabetic *INS*^C94Y^ Transgenic Pigs

After pathway interpretation of the proteome data from porcine CD4^+^ T cells, we subsequently focused on the analysis of single proteins. From the 80 significantly differing proteins, we were especially interested in two candidates with potential biological relevance (*INS*^C94Y^ tg/wt ratio ≥ 2), namely MYL4, a regulatory light chain of myosin that binds actin and regulates ATPase activity (27) and ANXA1 (**Supplementary Table 2**, **Figure 3B**). ANXA1, a phospholipid-binding protein, was previously described as a regulator of inflammatory cells with anti-inflammatory and pro-resolving properties (28). It is widely expressed in tissues and has been found in a soluble form in biological fluids (29). ANXA1 showed highest statistical significance in protein abundance differences between wild-types and diabetic pigs (***p < 0.001). Interestingly, higher levels of ANXA1 were also already described in serum and plasma of type 1 (30) and type 2 diabetes mellitus patients (31). Therefore, we subsequently focused our candidate analyses on ANXA1.

### ANXA1 Was Higher Abundant at CD4^+^ T Cell Outer Cell Membranes of Diabetic *INS*^C94Y^ Transgenic Pigs

**Figure 5:**
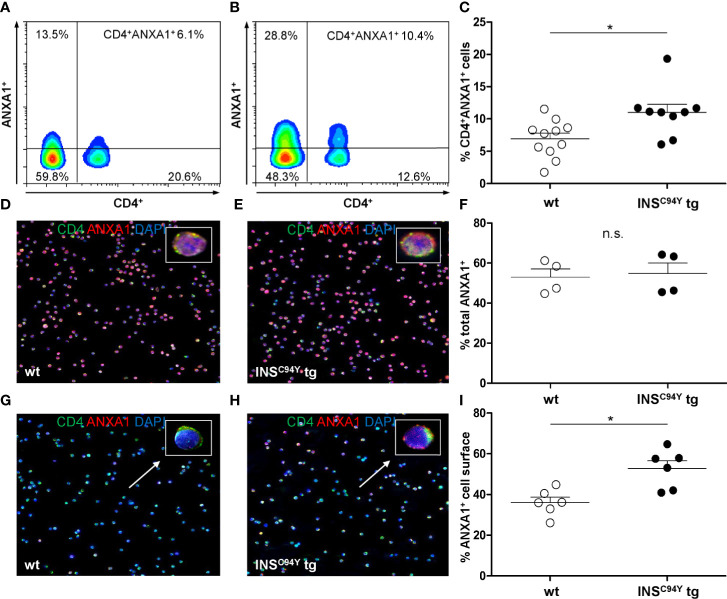
Differential ANXA1 expression in porcine CD4^+^ T cells. **(A–C)** Representative flow cytometric images of ANXA1^+^CD4^+^ T cells of a wild-type **(A)** and an *INS*^C94Y^ tg pig **(B)** are shown with reduced ANXA1 expression in wild-type CD4^+^ T cells. **(C)** Scatter plots summarizing flow cytometry analyses of PBMC of eleven wild-type and nine *INS*^C94Y^ tg pigs. ANXA1 was significantly elevated in CD4^+^ T cells of *INS*^C94Y^ tg pigs (*p < 0.05). **(D–F)** Similar ANXA1 levels after permeabilization in purified CD4^+^ T cells of a wild-type **(D)** and an *INS*^C94Y^ tg pig **(E)** shown by immunofluorescence staining for ANXA1 (red), CD4 (green) and nucleus (blue, counterstained with DAPI) and confirmed by flow cytometry using n=4 per group **(F)**. **(G–I)** Representative images of immunofluorescence staining for ANXA1 on the outer cell membrane of porcine CD4^+^ T cells demonstrate higher ANXA1 expression in CD4^+^ T cells of the *INS*^C94Y^ tg pig **(H)** compared to the wild-type **(G)** (arrows). **(I)** A higher ANXA1 expression on the cell surface of diabetic CD4^+^ T cells compared to CD4^+^ T cells of wild-types was confirmed by flow cytometry (n=6 per group). Horizontal bars indicate group means.

Flow cytometric analyses confirmed that ANXA1 expression was significantly increased in CD4^+^ T cells of diabetic *INS*^C94Y^ tg pigs (n=9) compared to wild-types (n=11) (*p < 0.05; **Figures 5A–C**; gating strategy: **Supplementary Figure 4**). To determine the subcellular location of ANXA1, we analyzed purified CD4^+^ T cells with immunocytology. While ANXA1 levels after permeabilization were equivalent between both groups (**Figures 5D–F**; n=4 per group), ANXA1 abundance was significantly enhanced at the outer cell membrane of CD4^+^ T cells from diabetic pigs (*p < 0.05; **Figures 5G–I**; n= 6 per group). Although expression of ANXA1 was also observed on other PBMC subpopulations (CD8α^+^ T cells, SWC5^+^ γδ T cells and CD79a^+^ B cells) no significant abundance difference of ANXA1 was evident in the non-CD4 cell fraction between *INS*^C94Y^ tg pigs (n=4) and wild-types (n=5) of the animals analyzed with differential proteomics (ratio 1.4, p-value 0.09).

## Discussion

To assess the impact of permanent hyperglycemia on immune cell function *in vivo*, we characterised lymphocytes of a transgenic pig model for permanent neonatal diabetes mellitus. We observed significant changes in body weight, blood glucose and serum insulin levels in these diabetic pigs at the age of 12 weeks. Compared to non-transgenic wild-type littermates, significant differences in proliferative response, CD4^+^ T cell proteomes and the metabolic phenotypes were already detectable in this early diabetic pig model. Our findings point to main distinct changes in the T cell compartment triggered by early-life high blood glucose and indicate that several immune alterations already occur in the early stage of diabetes mellitus.

Importantly, our study was performed with diabetic pigs lacking insulin supplementation. The stable diabetic phenotype with permanently high blood glucose and low serum insulin levels makes the *INS*^C94Y^ tg pig an attractive model for translational diabetes research. Since insulin is a major growth factor and anabolic hormone (32), body weight of diabetic pigs was markedly reduced compared to wild-type pigs due to lower growth rate, as previously described by Renner et al. (13). Investigating immunological properties of this diabetic pig model, we found no differences of lymphocyte proliferation between controls and diabetic pigs after polyclonal stimulation with B and T cell mitogen PWM and T cell mitogen ConA. However, after polyclonal stimulation with T cell mitogen PHA, we detected a distinct decreased proliferation of lymphocytes from diabetic pigs, which points to an impaired immune response selectively induced by this mitogen. This fits in well with our pathway analysis data, which showed distinct clustering of high abundant proteins from diabetic CD4^+^ T cells to immune system and signal transduction processes. Interestingly, a decreased response of T cells to PHA-stimulation was already demonstrated in diabetic patients (33, 34). The significantly reduced proliferation to PHA was correlated with a decreased interleukin (IL)-2 production (34). The cytokine IL-2, as well as its IL-2 receptor, is a cytokine crucial for proliferation processes in human T cells (35) and, thus, is enhanced in these cells after PHA stimulation leading to cell proliferation (36). These findings were supported and extended by another transcriptomic study of PHA-activated human CD4^+^ and CD8^+^ T cells with decreased gene expression of IL-2 and IL-2 receptor in diabetic type 2 patients (37). Furthermore, T cells from these patients exhibited differences in expression of additional genes and gene products like lower expression of genes of insulin signaling pathway and enzymes of the glycolytic pathway (37), indicating that an impaired immune response in the diabetic context induced by PHA is affected by more than IL-2 signaling. Little is known about the precise signaling pathways (37), and intracellular processes stimulated by PHA and ConA in humans and pigs. However, the impaired immune response selectively induced by PHA in hyperglycemic conditions, which is not addressed by polyclonal T cell mitogen ConA (22), points to PHA-associated cell signaling pathways specifically altered in the diabetic condition. In murine T cells, ConA was shown to bind to surface glycoproteins and with high affinity to the co-stimulatory molecule CD28, leading to enhanced T cell proliferation (38). If mechanisms hold true for porcine T cells, hyperglycemia does not affect CD28 signaling in diabetic pigs. In contrast, PHA is able to mimic the stimulation of T cells *via* crosslinking of T cell receptor and CD3 complex, which is followed by a sustained calcium influx as an important signal for T cell proliferation (23). In the Jurkat T cell line, high glucose levels were shown to inhibit T cell activation *via* an increase in non-enzymatic glycation of concentration-regulating calcium channels and delayed specific activation of these cells with anti-CD3 monoclonal antibody (39). Since alterations of calcium homeostasis were shown in various cell types of diabetic patients (40), insufficient calcium-mediated signaling in T cells of diabetic pigs might point to impaired PHA-induced proliferation. Thus, the immune cell calcium homeostasis of diabetic *INS*^C94Y^ tg pigs merits further investigations in future studies to examine possible correlations to the impaired T cell response in the diabetic cases observed in our study.

To gain deeper insights into hyperglycemia -associated molecular changes of CD4^+^ T cells and their impact on cell function, we analyzed the cell-derived proteome from diabetic pigs and wild-types as a hypothesis-generating approach for the dysregulated immune responses. Notably, we obtained a comprehensive proteomic data set for porcine CD4^+^ T cells with a high resolution of 2,704 proteins identified with ≥ 2 peptides. Among these, a total of 3% (80 proteins) showed significantly different protein abundances between groups, indicating distinct proteome differences in CD4^+^ T cells from early stage diabetic pigs. While many studies of immune cells from diabetic type 2 patients were performed by a 2D-gelelectrophoresis approach, only significantly altered spots were analysed subsequently with mass spectrometry (10, 41). To our knowledge, there is no in-depth proteomic fingerprint of CD4^+^ T cells in hyperglycemic conditions at initial stage of non-immune-mediated cases of diabetes mellitus. Interestingly, the protein with the highest statistical significance and a 2-fold higher abundance in diabetic pigs was ANXA1. This protein identification corresponds to findings in type 1 (30) and type 2 (31) diabetic patients, where ANXA1 was previously found with increased levels in serum and plasma, pointing to a pivotal, disease-associated alteration of this protein. However, the biological consequence of elevated ANXA1 in diabetes mellitus remains unexplained (28). While human and mouse neutrophils, monocytes and macrophages constitutively contain high levels of ANXA1 in their cytoplasm, T cells express the protein at lower levels (42). Following cell activation, ANXA1 is readily mobilized to the cell surface where it interacts with its receptor named formyl peptide receptor 2 (FPR2) in a paracrine/autocrine manner (43). Early studies in mice have shown that ANXA1 acts as a molecular T cell tuner by increasing the strength of T cell receptor signaling and T cell activation (44). Overexpressed ANXA1 induced the differentiation of naïve T cells to pro-inflammatory Th1 cells with a high expression of INFγ (45), promoting an inflammatory milieu. Thus, the higher abundance of ANXA1 on the surface of CD4^+^ T cells of diabetic pigs might indicate an enhanced T cell activation with functional importance of a pro-inflammatory immune response. However, also the opposite effect was shown in ANXA1-deficient mouse CD4^+^ T cells (46). These cells increased their activation, proliferation and inflammation in the absence of ANXA1, suggesting that this protein rather has an anti-inflammatory role and attenuates an exacerbated inflammatory response (46). One possible reason is that externalized ANXA1 regulates the extracellular regulated protein kinases (ERK)/mitogen-activated protein kinases (MAPK) signaling pathway *via* binding to its formyl peptide receptor (FPR) (30). This pathway is important for T cell activity and strongly contributes to T cell activation and proliferation (47) and was shown to be activated in hyperglycemia (30). Treatment of *Anxa1*^−/−^ mice with human recombinant ANXA1 attenuated inflammatory processes in tissues by reducing MAPK signaling (30). Thus, a higher abundance of ANXA1 in diabetic CD4^+^ T cells may temper signaling to protect the cell from hyperresponsiveness initiated by chronic hyperglycemia and, thus, exert anti-inflammatory effects. Although we cannot yet define the precise role of ANXA1 in CD4^+^ T cells in the porcine diabetic model, the identification of ANXA1 in humans and pigs, independent of etiology, is an important finding which underscores a crucial role of this protein in T cell impairment accompanying diabetes mellitus. It furthermore highlights the potential translational quality of this animal model for diabetes research, which is, in our opinion, recommended for future studies to examine the role of this molecule for the observed dysregulated immune responses.

Pathway analysis of our proteome dataset revealed distinctly different patterns of over-represented pathways between CD4^+^ T cells from diabetic pigs and wild-type littermates. Proteins with higher abundance in wild-types, hence lower abundance in diabetic pigs, mainly clustered to pathways involved in gene regulation, which may explain the decreased proliferation capacity observed in immune cells from diabetic pigs. Proteins with increased abundance in diabetic pigs, on the other hand, mainly associated to immune system, signal transduction and metabolic pathways. Interestingly, one of the most over-represented pathways associated to altered proteins in CD4^+^ T cells of diabetic pigs was lipophagy. Lipophagy is linked to metabolic dysfunctions of cells (25, 26), and thus, pointed to an altered metabolic phenotype in diabetic pigs. We therefore characterized the metabolic immune cell phenotype of PBMC, since cellular metabolism can be a primary driver and regulator of immune cell function (12). Metabolic properties of diabetic PBMC in this study revealed an increase in mitochondrial respiration and basal glycolytic activity. We found significant increases in mitochondrial oxygen consumption rate (OCR) in PBMC of diabetic pigs. Interestingly, in line with our findings, increased oxygen consumption was also demonstrated in PBMC of type 2 diabetes patients (48). Previously, this group also measured a higher production of reactive oxygen species (ROS) in these cells and hypothesized mitochondrial damage in diabetic PBMC caused by enhanced oxidative stress. An association of hyperglycemia to glucose-mediated increase in ROS may activate the “dangerous metabolic route in diabetes” through diacylglycerol (DAG), protein kinase C (PKC) and NADPH oxidase [reviewed in (49)]. This results in intracellular accumulation of ROS, which has cytotoxic effects on the cell, possibly affecting proliferation ability of cells, as described in our studies. Since ROS synthesis is essential for physiological immune activation of lymphocytes but lead to cellular damage if concentration surpasses (50), this parameter should be determined in PBMC of diabetic pigs, in order to evaluate whether immune cells are impaired by oxidative cell stress. On the contrary, mitochondrial respiration with increased oxygen consumption is used by immune cells to supplement increased glycolysis, which is the main metabolic pathway fueling effector function upon T cell activation (51). Besides increased oxygen consumption rates, we also found a higher basal glycolytic activity in early diabetic pigs, suggesting that hyperglycemia induced metabolic adaption to local conditions of immune cells and promoted a more activated status in T cells (52). A rapid increase in glycolysis is an important determinant in cellular energy production and required for effective cell proliferation, size expansion, and differentiation (51). Hence, it is also possible that PBMC in young diabetic pigs are highly metabolically active to challenge hyperglycemic conditions. Our findings indicate that metabolic disturbances occurring at early stage diabetes mellitus, as observed in *INS*^C94Y^ tg pigs, likely cause metabolic reprogramming in PBMC and may also influence their activation status. Since further studies are absolutely required for a better understanding of bioenergetic profiles of PBMC in diabetes mellitus, diabetic pigs enable to define mitochondrial properties and cellular energy production in these cells at initial stage of disease.

Taken together, our findings point to an early, fundamental dysregulation of immune cells when exposed to prolonged high blood glucose. Chronic hyperglycemia leads to profound molecular and functional changes of T cells reasoned by proteomic and metabolic alterations, and, moreover, affects T cell immune responses *in vitro*. Diabetic pigs qualify for additional translational experiments to explore the crucial role of altered ANXA1 levels in adaptive immunity in diabetes mellitus. The altered metabolic properties of immune cells in young diabetic pigs provided novel information on distinct immune cell metabolism at initial stage of disease. The exact meaning of this significantly altered metabolic phenotype of the immune cells is not known so far, thus, *INS*^C94Y^ tg pigs are a valuable source to further analyze respective changes for the diagnosis, or treatment or even prevention of early stage diabetes mellitus and its immune system-related complications.

## Data Availability Statement

The raw data supporting the conclusions of this article will be made available by the authors, without undue reservation.

## Ethics Statement

The animal study was reviewed and approved by the Government of Upper Bavaria.

## Author Contributions

CD conceived and designed the experiments. I-MG, RD, M-CS, MW, SH, and CD performed the experiments. I-MG, SH, M-CS, MW, and RD analyzed the data. SR and EW developed and characterized the *INS*^C94Y^ tg pig model and contributed the reagents and materials. I-MG and CD wrote the manuscript. All co-authors critically read the manuscript and approved the final version to be published. CD is the guarantor of this work and, as such, had full access to all the data in the study and takes responsibility for the integrity of the data and the accuracy of the data analysis. All authors contributed to the article and approved the submitted version.

## Funding

This work was supported by grants from the Deutsche Forschungsgemeinschaft SPP project 2127 (DFG DE 719/7-1 to CD, HA 6014/5-1 to SH) and by the German Center for Diabetes Research (82DZD00802 to EW and SR).

## Conflict of Interest

The authors declare that the research was conducted in the absence of any commercial or financial relationships that could be construed as a potential conflict of interest.

## References

[1] Harding JL Pavkov ME Magliano DJ Shaw JE Gregg EW Global trends in diabetes complications: a review of current evidence. Diabetologia (2019) 62(1):3–16. 10.1007/s00125-018-4711-2 30171279

[2] Kumar NP Fukutani KF Shruthi BS Alves T Silveira-Mattos PS Rocha MS Persistent inflammation during anti-tuberculosis treatment with diabetes comorbidity. Elife (2019) 8:1–19. 10.7554/eLife.46477 PMC6660216 31271354

[3] Marshall RJ Armart P Hulme KD Chew KY Brown AC Hansbro PM Glycemic Variability in Diabetes Increases the Severity of Influenza. mBio (2020) 11(2):1–15. 10.1128/mBio.02841-19 PMC7157527 32209691

[4] Zhou F Yu T Du R Fan G Liu Y Liu Z Clinical course and risk factors for mortality of adult inpatients with COVID-19 in Wuhan, China: a retrospective cohort study. Lancet (2020) 395(10229):1054–62. 10.1016/S0140-6736(20)30566-3 PMC7270627 32171076

[5] Hu Y Sun J Dai Z Deng H Li X Huang Q Prevalence and severity of corona virus disease 2019 (COVID-19): A systematic review and meta-analysis. J Clin Virol (2020) 127:104371. 10.1016/j.jcv.2020.104371 32315817 PMC7195434

[6] Nicholas DA Proctor EA Agrawal M Belkina AC Van Nostrand SC Panneerseelan-Bharath L Fatty Acid Metabolites Combine with Reduced β Oxidation to Activate Th17 Inflammation in Human Type 2 Diabetes. Cell Metab (2019) 30(3):447–61.e5. 10.1016/j.cmet.2019.07.004 31378464 PMC8506657

[7] Nyambuya TM Dludla PV Mxinwa V Nkambule BB. T-cell activation and cardiovascular risk in adults with type 2 diabetes mellitus: A systematic review and meta-analysis. Clin Immunol (Orlando Fla) (2020) 210:108313. 10.1016/j.clim.2019.108313 31765833

[8] Lee Y-h Kim SR Han DH Yu HT Han YD Kim JH Senescent T Cells Predict the Development of Hyperglycemia in Humans. Diabetes (2019) 68(1):156. 10.2337/db17-1218 30389747

[9] Lau EYM Carroll EC Callender LA Hood GA Berryman V Pattrick M Type 2 diabetes is associated with the accumulation of senescent T cells. Clin Exp Immunol (2019) 197(2):205–13. 10.1111/cei.13344 PMC6642873 31251396

[10] Soongsathitanon J Umsa-Ard W Thongboonkerd V Proteomic analysis of peripheral blood polymorphonuclear cells (PBMCs) reveals alteration of neutrophil extracellular trap (NET) components in uncontrolled diabetes. Mol Cell Biochem (2019) 461(1-2):1–14. 10.1007/s11010-019-03583-y 31273604

[11] Lepper MF Ohmayer U von Toerne C Maison N Ziegler AG Hauck SM Proteomic Landscape of Patient-Derived CD4+ T Cells in Recent-Onset Type 1 Diabetes. J Proteome Res (2018) 17(1):618–34. 10.1021/acs.jproteome.7b00712 29182335

[12] Olenchock BA Rathmell JC Vander Heiden MG Biochemical Underpinnings of Immune Cell Metabolic Phenotypes. Immunity (2017) 46(5):703–13. 10.1016/j.immuni.2017.04.013 PMC5660630 28514672

[13] Renner S Braun-Reichhart C Blutke A Herbach N Emrich D Streckel E Permanent neonatal diabetes in INS(C94Y) transgenic pigs. Diabetes (2013) 62(5):1505–11. 10.2337/db12-1065 PMC3636654 23274907

[14] Hinkel R Howe A Renner S Ng J Lee S Klett K Diabetes Mellitus-Induced Microvascular Destabilization in the Myocardium. J Am Coll Cardiol (2017) 69(2):131–43. 10.1016/j.jacc.2016.10.058 28081822

[15] Kleinwort KJH Amann B Hauck SM Hirmer S Blutke A Renner S Retinopathy with central oedema in an INS (C94Y) transgenic pig model of long-term diabetes. Diabetologia (2017) 60(8):1541–9. 10.1007/s00125-017-4290-7 28480495

[16] Czajka A Ajaz S Gnudi L Parsade CK Jones P Reid F Altered Mitochondrial Function, Mitochondrial DNA and Reduced Metabolic Flexibility in Patients With Diabetic Nephropathy. EBioMedicine (2015) 2(6):499–512. 10.1016/j.ebiom.2015.04.002 26288815 PMC4534759

[17] Grosche A Hauser A Lepper MF Mayo R von Toerne C Merl-Pham J The Proteome of Native Adult Muller Glial Cells From Murine Retina. Mol Cell Proteomics MCP (2016) 15(2):462–80. 10.1074/mcp.M115.052183 PMC4739667 26324419

[18] Singh J Kaade E Muntel J Bruderer R Reiter L Thelen M Systematic Comparison of Strategies for the Enrichment of Lysosomes by Data Independent Acquisition. J Proteome Res (2020) 19(1):371–81. 10.1021/acs.jproteome.9b00580 31738065

[19] Reiter L Rinner O Picotti P Huttenhain R Beck M Brusniak MY mProphet: automated data processing and statistical validation for large-scale SRM experiments. Nat Methods (2011) 8(5):430–5. 10.1038/nmeth.1584 21423193

[20] van der Windt GJW Chang CH Pearce EL Measuring Bioenergetics in T Cells Using a Seahorse Extracellular Flux Analyzer. Curr Protoc Immunol (2016) 113:3.16b.1–3.b.4. 10.1002/0471142735.im0316bs113 PMC4864360 27038461

[21] Nam JH Cha B Park JY Abekura F Kim CH Kim JR Mitogen-Induced Interferon Gamma Production in Human Whole Blood: The Effect of Heat and Cations. Curr Pharm Biotechnol (2019) 20(7):562–72. 10.2174/1389201020666190528093432 31132974

[22] Rodríguez-Gómez IM Talker SC Käser T Stadler M Reiter L Ladinig A Expression of T-Bet, Eomesodermin, and GATA-3 Correlates With Distinct Phenotypes and Functional Properties in Porcine γδ T Cells. Front Immunol (2019) 10(396):1-21. 10.3389/fimmu.2019.00396 30915070 PMC6421308

[23] Lin VH-C Chen J-J Liao C-C Lee S-S Chien EJ The rapid immunosuppression in phytohemagglutinin-activated human T cells is inhibited by the proliferative Ca2+ influx induced by progesterone and analogs. Steroids (2016) 111:71–8. 10.1016/j.steroids.2016.01.010 26808612

[24] Jassal B Matthews L Viteri G Gong C Lorente P Fabregat A The reactome pathway knowledgebase. Nucleic Acids Res (2020) 48(D1):D498–503. 10.1093/nar/gkz1031 PMC7145712 31691815

[25] Kounakis K Chaniotakis M Markaki M Tavernarakis N Emerging Roles of Lipophagy in Health and Disease. Front Cell Dev Biol (2019) 7:185(185):1–8. 10.3389/fcell.2019.00185 31552248 PMC6746960

[26] Tian Y Yang B Qiu W Hao Y Zhang Z Yang B ER-residential Nogo-B accelerates NAFLD-associated HCC mediated by metabolic reprogramming of oxLDL lipophagy. Nat Commun (2019) 10(1):3391. 10.1038/s41467-019-11274-x 31358770 PMC6662851

[27] Ho G Chisholm RL Substitution mutations in the myosin essential light chain lead to reduced actin-activated ATPase activity despite stoichiometric binding to the heavy chain. J Biol Chem (1997) 272(7):4522–7. 10.1074/jbc.272.7.4522 9020178

[28] Purvis GSD Solito E Thiemermann C Annexin-A1: Therapeutic Potential in Microvascular Disease. Front Immunol (2019) 10:938:938. 10.3389/fimmu.2019.00938 31114582 PMC6502989

[29] Pietrani NT Ferreira CN Rodrigues KF Perucci LO Carneiro FS Bosco AA Proresolving protein Annexin A1: The role in type 2 diabetes mellitus and obesity. Biomed Pharmacother Biomed Pharmacother (2018) 103:482–9. 10.1016/j.biopha.2018.04.024 29677533

[30] Purvis GSD Chiazza F Chen J Azevedo-Loiola R Martin L Kusters DHM Annexin A1 attenuates microvascular complications through restoration of Akt signalling in a murine model of type 1 diabetes. Diabetologia (2018) 61(2):482–95. 10.1007/s00125-017-4469-y PMC6448955 29085990

[31] Purvis GSD Collino M Loiola RA Baragetti A Chiazza F Brovelli M Identification of AnnexinA1 as an Endogenous Regulator of RhoA, and Its Role in the Pathophysiology and Experimental Therapy of Type-2 Diabetes. Front Immunol (2019) 10:571:571. 10.3389/fimmu.2019.00571 30972066 PMC6446914

[32] van Niekerk G Christowitz C Conradie D Engelbrecht AM Insulin as an immunomodulatory hormone. Cytokine Growth Factor Rev (2019) 52:34-44. 10.1016/j.cytogfr.2019.11.006 31831339

[33] Chang FY Shaio MF Decreased cell-mediated immunity in patients with non-insulin-dependent diabetes mellitus. Diabetes Res Clin Pract (1995) 28(2):137–46. 10.1016/0168-8227(95)00168-8 7587921

[34] Richard C Wadowski M Goruk S Cameron L Sharma AM Field CJ Individuals with obesity and type 2 diabetes have additional immune dysfunction compared with obese individuals who are metabolically healthy. BMJ Open Diabetes Res Care (2017) 5(1):e000379. 10.1136/bmjdrc-2016-000379 PMC5530252 28761653

[35] Abbas AK Trotta E Simeonov DR Marson A Bluestone JA Revisiting IL-2: Biology and therapeutic prospects. Sci Immunol (2018) 3(25):1–8. 10.1126/sciimmunol.aat1482 29980618

[36] Hu S Chen CW Chen ST Tsui KH Tang TK Cheng HT Inhibitory effect of berberine on interleukin-2 secretion from PHA-treated lymphocytic Jurkat cells. Int Immunopharmacol (2019) 66:267–73. 10.1016/j.intimp.2018.11.020 30502647

[37] Stentz FB Kitabchi AE Transcriptome and Proteome Expressions Involved in Insulin Resistance in Muscle and Activated T-Lymphocytes of Patients with Type 2 Diabetes. Genom Proteomics Bioinf (2007) 5(3):216–35. 10.1016/S1672-0229(08)60009-1 PMC5054231 18267303

[38] Ando Y Yasuoka C Mishima T Ikematsu T Uede T Matsunaga T Concanavalin A-mediated T cell proliferation is regulated by herpes virus entry mediator costimulatory molecule. Vitro Cell Dev Biol Anim (2014) 50(4):313–20. 10.1007/s11626-013-9705-2 24163161

[39] Boldizsár F Berki T Miseta A Németh P Effect of hyperglycemia on the basal cytosolic free calcium level, calcium signal and tyrosine-phosphorylation in human T-cells. Immunol Lett (2002) 82(1-2):159–64. 10.1016/s0165-2478(02)00032-9 12008048

[40] Klec C Ziomek G Pichler M Malli R Graier WF Calcium Signaling in ß-cell Physiology and Pathology: A Revisit. Int J Mol Sci (2019) 20(24):6110. 10.3390/ijms20246110 PMC6940736 31817135

[41] Giorgi A Tempera I Napoletani G Drovandi D Potestà C Martire S Poly(ADP-ribosylated) proteins in mononuclear cells from patients with type 2 diabetes identified by proteomic studies. Acta Diabetol (2017) 54(9):833–42. 10.1007/s00592-017-1013-y 28608282

[42] Gavins FN Hickey MJ Annexin A1 and the regulation of innate and adaptive immunity. Front Immunol (2012) 3:354:354. 10.3389/fimmu.2012.00354 23230437 PMC3515881

[43] Perretti M D’Acquisto F Annexin A1 and glucocorticoids as effectors of the resolution of inflammation. Nat Rev Immunol (2009) 9(1):62–70. 10.1038/nri2470 19104500

[44] D’Acquisto F Merghani A Lecona E Rosignoli G Raza K Buckley CD Annexin-1 modulates T-cell activation and differentiation. Blood (2007) 109(3):1095–102. 10.1182/blood-2006-05-022798 PMC1855438 17008549

[45] Huang P Zhou Y Liu Z Zhang P Interaction between ANXA1 and GATA-3 in Immunosuppression of CD4(+) T Cells. Mediators Inflammation (2016) 2016:1701059. 10.1155/2016/1701059 PMC5090097 27833268

[46] Yang YH Song W Deane JA Kao W Ooi JD Ngo D Deficiency of annexin A1 in CD4+ T cells exacerbates T cell-dependent inflammation. J Immunol (Baltimore Md 1950) (2013) 190(3):997–1007. 10.4049/jimmunol.1202236 23267026

[47] Liu J Guo K Hu L Luo T Ma Y Zhang Y ZAP70 deficiency promotes reverse cholesterol transport through MAPK/ERK pathway in Jurkat cell. Mol Immunol (2019) 107:21–8. 10.1016/j.molimm.2019.01.001 30639475

[48] Hartman ML Shirihai OS Holbrook M Xu G Kocherla M Shah A Relation of mitochondrial oxygen consumption in peripheral blood mononuclear cells to vascular function in type 2 diabetes mellitus. Vasc Med (London England) (2014) 19(1):67–74. 10.1177/1358863x14521315 PMC3932629 24558030

[49] Volpe CMO Villar-Delfino PH Dos Anjos PMF Nogueira-Machado JA Cellular death, reactive oxygen species (ROS) and diabetic complications. Cell Death Dis (2018) 9(2):119. 10.1038/s41419-017-0135-z 29371661 PMC5833737

[50] Alfatni A Riou M Charles AL Meyer A Barnig C Andres E Peripheral Blood Mononuclear Cells and Platelets Mitochondrial Dysfunction, Oxidative Stress, and Circulating mtDNA in Cardiovascular Diseases. J Clin Med (2020) 9(2):1–24. 10.3390/jcm9020311 PMC7073649 31979097

[51] Gaber T Chen Y Krauss PL Buttgereit F Metabolism of T Lymphocytes in Health and Disease. Int Rev Cell Mol Biol (2019) 342:95–148. 10.1016/bs.ircmb.2018.06.002 30635095

[52] Bonacina F Baragetti A Catapano AL Norata GD The Interconnection Between Immuno-Metabolism, Diabetes, and CKD. Curr Diabetes Rep (2019) 19(5):21. 10.1007/s11892-019-1143-4 30888513

[53] Giese I-M Renner S Wolf E Hauck SM Deeg CA Chronic hyperglycaemia drives functional impairment of lymphocytes in diabetic *INS*^C94Y^ transgenic pigs [Preprint]. bioRxiv (2020). 10.1101/2020.08.26.267914 PMC7862560 33552065

