# Supplementary material for: Chronic Hyperglycemia Drives Functional Impairment of Lymphocytes in Diabetic INSC94Y Transgenic Pigs
Source: Front Immunol. 2021 Jan 22;11:607473. doi: 10.3389/fimmu.2020.607473 (PMC7862560; doi:10.3389/fimmu.2020.607473)
Supplement: Supplementary file 1 [file DataSheet_1.zip › Supplementary Figure 1.DOCX]

Supplementary Material


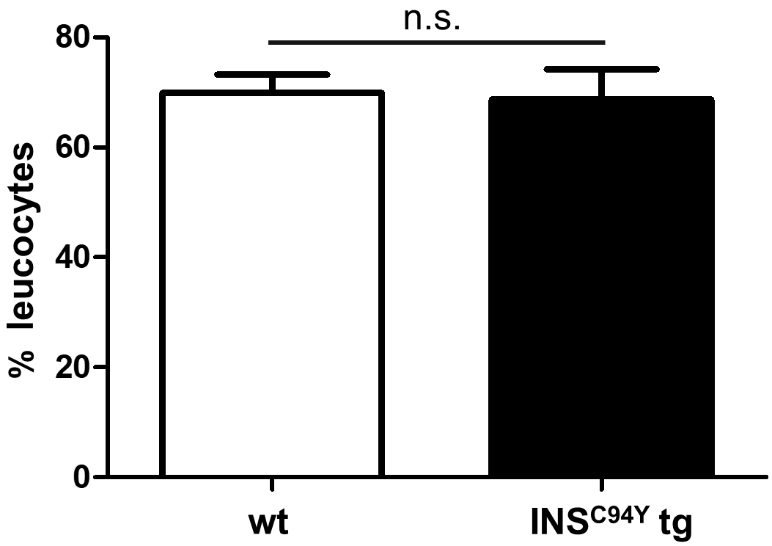


**Supplementary Fig. 1: Relative numbers of peripheral blood mononuclear cells counted via DiffQuick stained blood smears (A)** wt (n=6) and INS^C94Y^ tg pigs (n=6) displayed no significant difference in relative PBMC counts.
