# Supplementary material for: Chronic Hyperglycemia Drives Functional Impairment of Lymphocytes in Diabetic INSC94Y Transgenic Pigs
Source: Front Immunol. 2021 Jan 22;11:607473. doi: 10.3389/fimmu.2020.607473 (PMC7862560; doi:10.3389/fimmu.2020.607473)
Supplement: Supplementary file 1 [file DataSheet_1.zip › Supplementary Figure 2.DOCX]

Supplementary Material


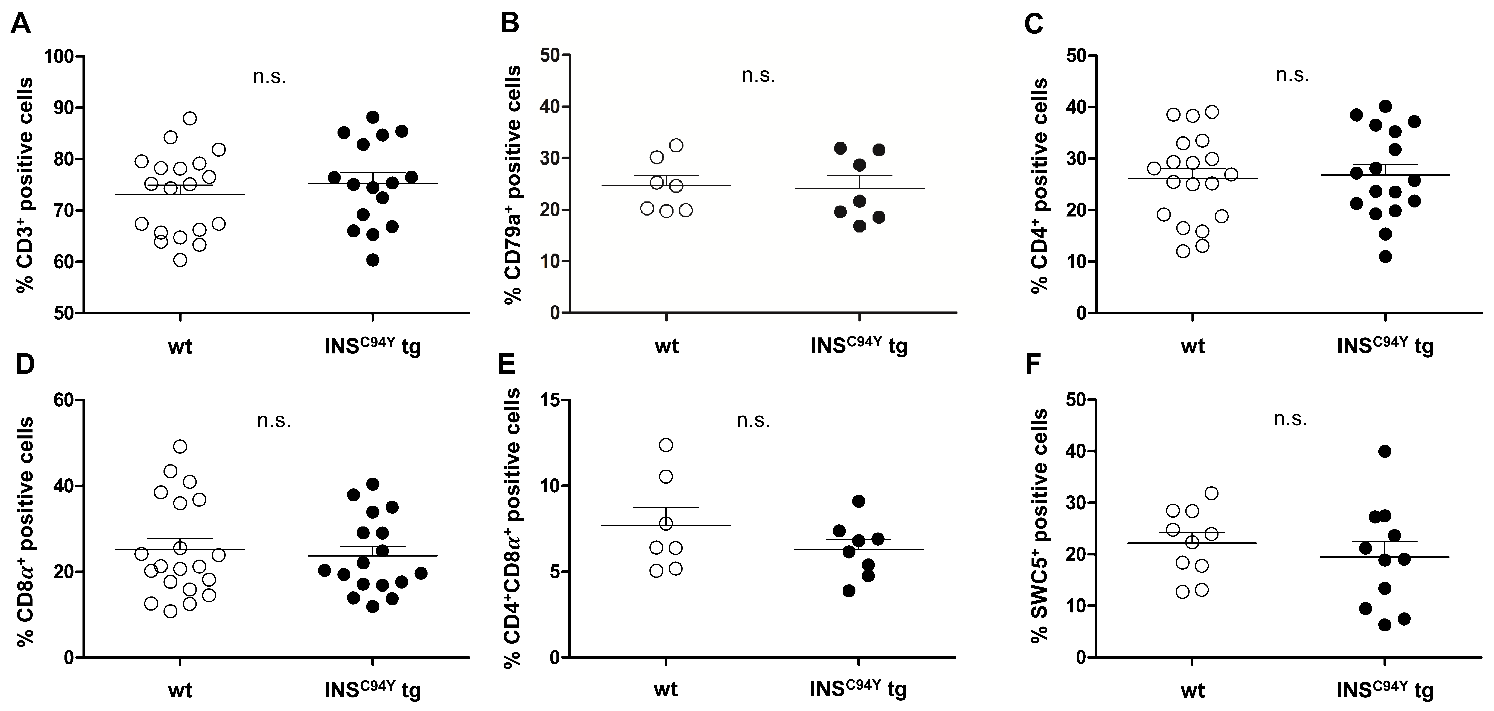


**Supplementary Fig. 2:** Lymphocyte subpopulations in porcine PBMC. Scatter plots illustrate percentages of lymphocyte subpopulations in wild-types and INS^C94Y^ tg pigs. Flow cytometry analyses revealed no significant differences in the percentage of CD3^+^ T cells (wt n=19; tg n=16) **(A)** and CD79a^+^ B cells (wt n=7; tg n=7) **(B)** and furthermore no significant differences in T cell subsets CD4^+^ (wt n=19; tg n=17) **(C)**, CD8α^+^ (wt n=20; tg n=17) **(D)**, CD4^+^CD8α^+^ (wt n=7; tg n=8) **(E)** and SWC5^+^ γδ T cells (wt n=10; tg n=11) **(F)**.
