# Supplementary material for: Chronic Hyperglycemia Drives Functional Impairment of Lymphocytes in Diabetic INSC94Y Transgenic Pigs
Source: Front Immunol. 2021 Jan 22;11:607473. doi: 10.3389/fimmu.2020.607473 (PMC7862560; doi:10.3389/fimmu.2020.607473)
Supplement: Supplementary file 1 [file DataSheet_1.zip › Supplementary Figure 3.DOCX]

Supplementary Material


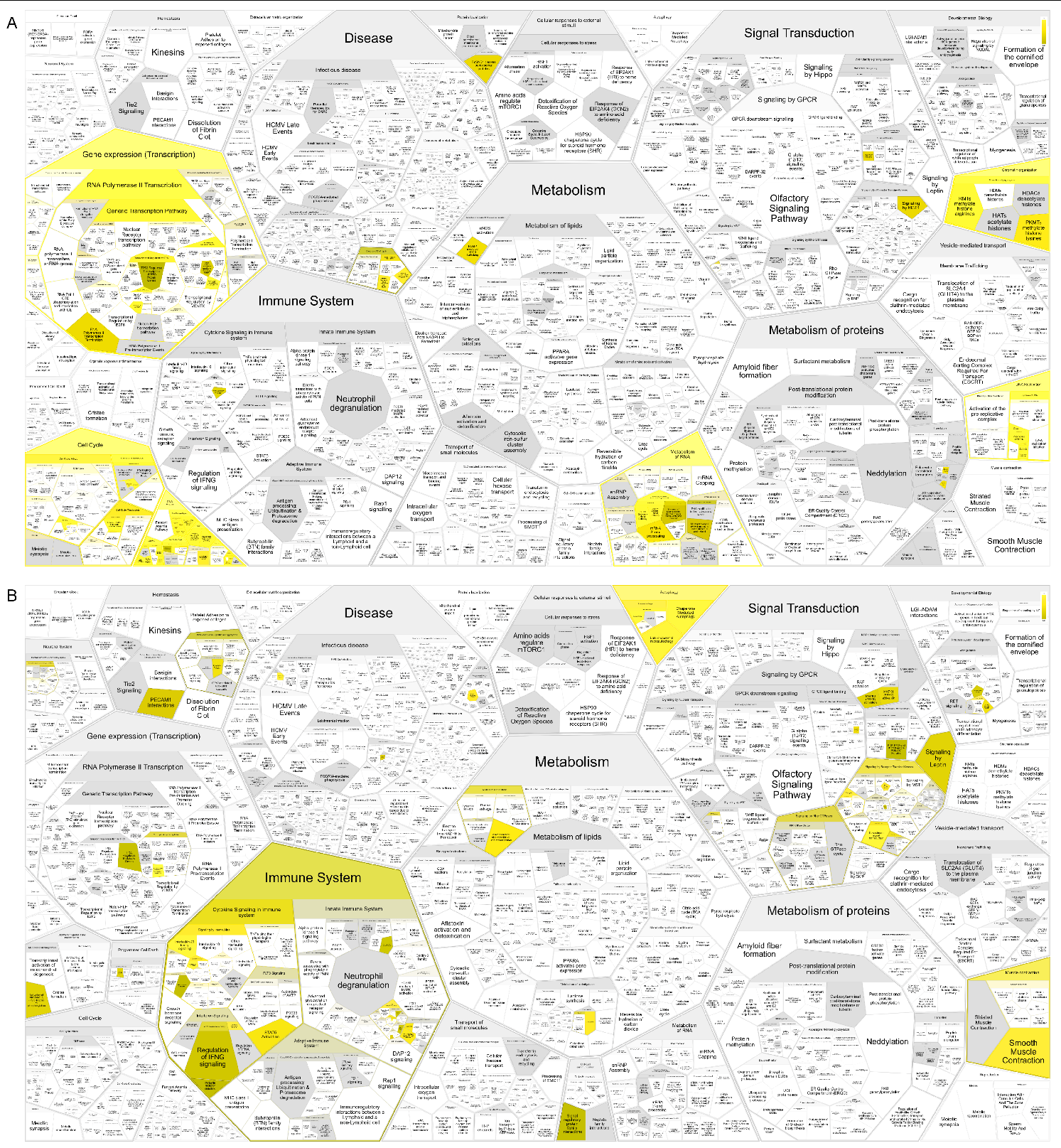


**Supplementary Fig. 3:** Overview of the Reactome analysis results pictured by Voronoi diagram comparing enriched pathways in CD4^+^ T cells of wild-types **(A)** and INS^C94Y^ tg pigs **(B)**. Pathway enrichment analysis was calculated with human orthologue gene names of proteins with significant (p < 0.05) abundance differences (A: ratio INS^C94Y^/wt < 1; B: ratio INS^C94Y^/wt > 1). Color brightness displays p-value of the statistical test for over-representation as illustrated by the color bar in the top right corner. Grey polygons show pathways which are not significantly over-represented. White areas represent pathways with no assigned proteins.
