# Supplementary material for: Chronic Hyperglycemia Drives Functional Impairment of Lymphocytes in Diabetic INSC94Y Transgenic Pigs
Source: Front Immunol. 2021 Jan 22;11:607473. doi: 10.3389/fimmu.2020.607473 (PMC7862560; doi:10.3389/fimmu.2020.607473)
Supplement: Supplementary file 1 [file DataSheet_1.zip › Supplementary Figure 4.DOCX]

Supplementary Material


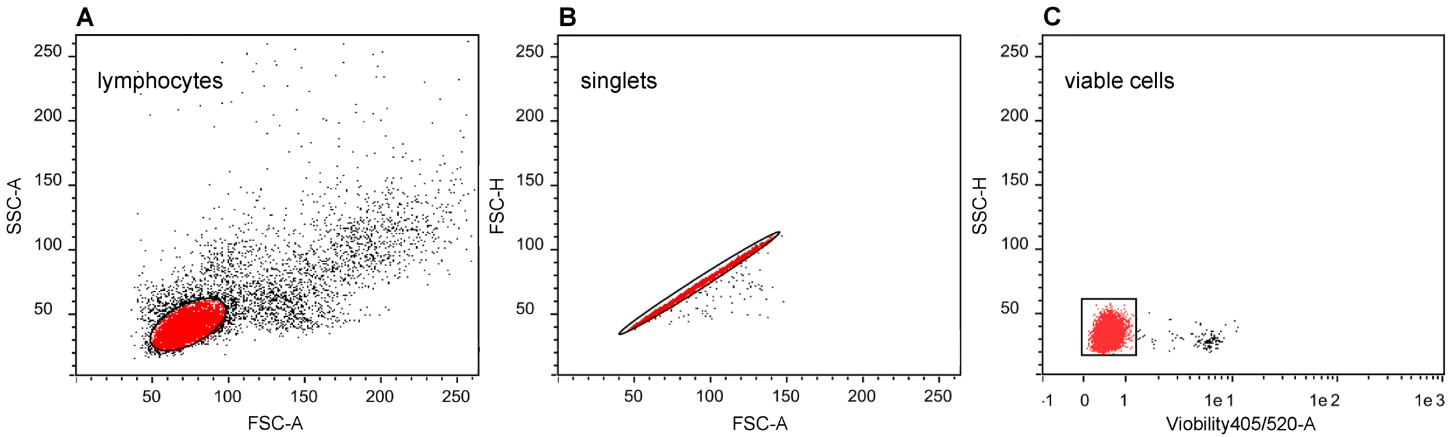


**Supplementary Fig. 4:** Gating strategy for flow cytometry experiments. PBMC of diabetic INS^C94Y^ tg pigs and wild-type littermates were isolated from whole blood via density gradient centrifugation, stained with Viobilty 405/520 Fixable Dye (Miltenyi Biotech) and measured with MACSQuant Analyzer 10 (Miltenyi Biotech). **(A)** Lymphocytes were gated according to size (FSC) and intracellular granularity (SSC). **(B)** doublets were excluded from lymphocyte population and **(C)** only viable singlets were included in further measurements.
